# End-User Feedback on a Low-Cost Portable Air Quality Sensor System—Are We There Yet?

**DOI:** 10.3390/s18113768

**Published:** 2018-11-04

**Authors:** Johanna Amalia Robinson, David Kocman, Milena Horvat, Alena Bartonova

**Affiliations:** 1 Department of Environmental Sciences, Jožef Stefan Institute, 1000 Ljubljana, Slovenia; david.kocman@ijs.si (D.K.); milena.horvat@ijs.si (M.H.); 2 Jožef Stefan International Postgraduate School, 1000 Ljubljana, Slovenia; 3 NILU—Norwegian Institute for Air Research, 2007 Kjeller, Norway; alena.bartonova@nilu.no

**Keywords:** low-cost portable sensor system, user experience, user needs, air quality, citizen science

## Abstract

Low-cost sensors are a current trend in citizen science projects that focus on air quality. Until now, devices incorporating such sensors have been tested primarily for their technical capabilities and limitations, whereas their usability and acceptability amongst the public rarely goes beyond proof of concept, leaving user experience (UX) unstudied. The authors argue that UX should be taken into account to make sure that products and services are fit for purpose. Nineteen volunteers tested and evaluated a prototype device and provided feedback through semi-structured interviews and during focus group sessions. Their UX was then coded using mixed coding methods regarding device functionality and recommendations for future product development. The results indicate that UX can identify potentially problematic design aspects while giving deeper insights into user needs. For example, UX recognized that one of the most important aspects of user involvement and motivation was successful data harvesting, which frequently failed. This study recommends that future developers of low-cost portable air quality sensor systems prioritize reliable data transmission to minimize data loss. This will ensure an efficient and positive UX that supports user engagement in citizen science based research where collecting sensor-based data is the primary objective.

## 1. Introduction

An increasing number of research projects requiring the use of portable low-cost air quality sensor systems [1] means there is a need for user experience (UX) feedback to sensor systems developers, and to those who deploy them. Our central argument is that developers of low-cost, portable air quality sensor systems and the scientists deploying them in participatory studies will benefit from considering end-user experience. Moreover, the trend in miniaturizing and mobilizing air quality sensor devices has influenced rapid development in the field of low-cost portable air quality sensor systems. In recent years, a large number of sensor developers and users have emerged, including commercial and research laboratories, reflecting the appeal of these devices for engaging various end users. There now exists a number of field studies characterizing such devices [2,3,4,5,6,7,8,9,10] including studies that explore their feasibility for personal exposure assessment [4,11,12,13,14]. Similarly, low-cost sensing devices utilizing IoT platforms designed for real–time indoor monitoring have been published [15,16,17,18,19] with indoor air quality being an important part of a holistic approach to exposure assessment. Many studies now focus on citizen science type experiments where volunteers carry or host an air quality sensor device [3,7,20,21,22].

Citizen science approaches have led to innovative methods of data collection, data processing, and data sharing both as a result of technological advances and an increased interest by the public to participate in gathering information about their environment [12,21,22,23,24]. Even if low-cost portable sensors can bring new opportunities for observing air pollution at higher spatial resolutions while providing personalized air quality data [25], there are many challenges preventing the wide-scale implementation of such devices. Some of these challenges relate to low-quality data deriving from the use of low-cost sensors and the lack of meaningful information that they provide to the public [3,8,21,26,27,28,29]. It is also recognized that although the involvement of citizens increases spatiotemporal coverage of an area [30], data collected by non-experts might be considered less reliable [31].

The added complexity created by involving citizens also adds to the challenges for the practical application of the technology. This problem has been addressed in several studies: For instance, Morawska et al. [32] provided an overview of the steps involved in successfully deploying sensor systems, whilst Thompson [22] pointed out a new technology should not be expected to work flawlessly, and encouraged researchers to develop not only accurate devices but to test them prior to deployment. Lewis et al. [7] also recommended caution when using newly developed sensor technologies and to first demonstrated their fitness for purpose, and Kumar et al. [26] raised questions about the future directions of low-cost sensors. In addition, Aoki et al. [25] questioned why participatory sensing is treated merely as a technical feasibility experiment, leaving the social aspect unstudied, whilst Hubbell [33] provided examples of what should be done and called for more social science research at the individual and community level.

Many authors e.g., [25,27,34,35,36], and review papers [21,22], have touched upon the user side of the technology. Most studies involving participants or studies describing a sensor-based solution for participatory sensing, however, discuss data processing, data transfer and data visualization [37,38,39,40], while few studies focus on the response to the information gathered, and how it can increase awareness and foster behavioral change [14,27,41]. Despite this, there remains a lack of literature from the perspectives of volunteers of systems which have not been designed with the potential end user in minds, yet have been deployed in citizen science research. Bales et al. [34,37] and Zappi et al. [36], as well as Willet et al. [35], provide examples of air quality sensing solution where the potential end users were iteratively involved in the development process, but these studies lack detailed insights.

Hart and Martinez raised usability issues as a challenge back in 2006 [42], and how sensor system networks are designed for research platforms used solely by scientists, while Moore [43] emphasized how high-tech products must meet end-users’ needs. However, devices reported in literature (see for example [8]) are still designed for those who have capabilities to perform co-location and calibration of the units, e.g., technically capable members of the public, professional communities or scientists. Furthermore, scientists often recruit the public to use a research device they consider as a “finished” product, yet in reality the device is a prototype. The present trend is to create user-friendly sensor systems for public use, yet there is lack of literature validating those claims. It is surprising how few sensor systems have been evaluated by potential end users. Studies show that the more the end user is involved in the process of designing and testing a product, the more satisfied they are with the final product as summarized by Mahmood et al. in their meta-analysis [44]. Commodore et al. [21] confirmed the importance of community involvement in all stages of the research process in the field of air quality related participatory research, and Reed [45] emphasizes that participation should start as early as possible in the design phase and involve relevant stakeholders to provide a comprehensive understanding of societal needs.

Without end-user involvement, it can be difficult for a designer to know intuitively what is required from a product. The benefits of learning about user behavior, needs and motivations through UX research far outweigh the risk of failure when users are not involved in the design process [46]. A key concept of citizens adapting new technology is “ease of use”. Scientist can study these aspects through usability testing, based on the landscape of Human-Centered design domain [47,48]. Documenting the interactional experience of the end user, can lighten technically oriented designers who commonly see the failure of the user to use the product correctly as the users fault e.g., by using Problem Exists Between Keyboard And Chair (PEBKAC)-type phrases. A human centered design approach can help make useful and user-friendly products [49] and contributing products that are fit for purpose.

This paper, provides a close examination of UX in relation to a prototype of a portable low-cost air quality sensor system based on a field study conducted within the EU funded CITI-SENSE project [50]. Our aim was to describe the outcomes of UX study regarding a product which would have otherwise not been tested by potential end users and hence identify potential improvements. To do this, volunteers were each given a device for one week to test and evaluate, after which they provided feedback through semi-structured interviews and focus group sessions. The feedback was then coded to obtain a structured summary of their experiences. The study maintained a constant discussion between researchers and the development team, and the volunteers’ feedback was provided promptly to the development team in order to contribute to the next generation of sensor systems. Ultimately, this paper discusses the types of features that the citizen science community desires, i.e., user needs as well as the pitfalls in design, which developers can avoid in order to create a positive user experience.

## 2. Methods

### 2.1. Study Design

Nineteen volunteers were recruited to test and evaluate a prototype low-cost portable sensor system in Ljubljana, Slovenia between 21 April 2016 and 24 June 2016. One sensor system was given to each of the volunteers for one week. The purpose of the study was to have volunteers to test and evaluate the sensor system during their daily routine rather than in a usability laboratory. As the sensors in the device were targeting outdoor air pollutants, the volunteers were recommended to carry the device on them when leaving the house, therefore they were not requested to carry the sensor unit at all times as is usually the case in exposure studies. Out of the 19 volunteers, 67% were male, and 37% female. 5% had finished secondary education as their highest level of education, 37% had an undergraduate degree, 5% had a master’s degree, and the remainder 53% had a doctoral degree. Ages ranged between 25 and 57 with 26% under 30 years old, 53% between 31 and 40 years old, and 21% were 41 years old or older. Volunteers included representatives from local technology-based businesses and national organizations, i.e., individuals with a better than expected average technological background, and individuals with a research background. This may have introduced some bias into the study, since the group of volunteers is not representative of the public. Initially, the study recruited public volunteers at the yearly Jožef Stefan institute’s open day event, and local stakeholders during project meetings. Other volunteers contacted the research team after they saw a presentation of the project (short-video) by the Slovenian Press Agency in their news feed. No incentives for participation were given, and all volunteers signed a user agreement and a privacy policy document.

Volunteer training involved a demonstration of how to use of the device during a face-to-face session. Each volunteer also received a link to a video tutorial [51] and a user guide [52] describing the use of the device, the main functionalities as well as troubleshooting including instructions on how to charge the device. Throughout the trial, volunteers had access to on-demand technical support by phone and email with the project researchers. All technical issues encountered were reported to the development team during the participation phase in order for the team to make the necessary adjustments. After the field trial, researchers provided a summary of the feedback to the development team.

The field trial was followed by UX research to gain an in-depth understanding of user needs and from the analysis of user feedback to systematically describe what worked well and what improvements are needed. The project team conducted six focus group sessions with volunteer numbers ranging from two to five volunteers. In addition, they conducted two open ended individual interviews with those volunteers who could not join the focus groups, one of them through an online GoToMeeting. The focus groups and interviews followed the same semi-structured form and were conducted at the volunteers’ workplace and moderated by the lead author. Volunteers were told in advance that they are expected to provide their feedback of the sensor system, however, they were not given any questions in advance. Face-to-face feedback sessions lasted approximately 20 min and the focus group sessions and interviews were recorded and transcribed. Four of the sessions were held in the Slovene language and were translated into English after transcription. The information gained provided feedback to the development team and research material for the current paper.

### 2.2. Low-Cost Portable Sensor Unit with Smartphone Visualization

**Figure 1:**
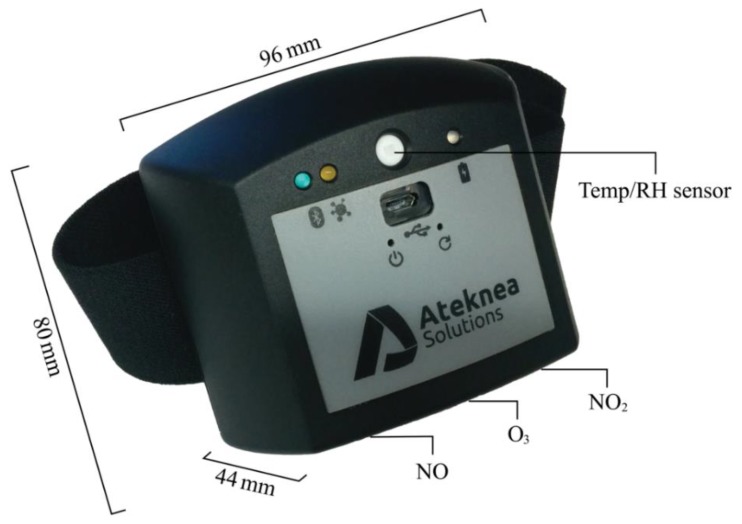
Little Environmental Observatory (LEO) portable sensor system.

This study, made use of a prototype low-cost portable air quality sensor system developed within the CITI-SENSE project (2012–2016). CITI-SENSE also developed hardware and software sensing platforms, which allowed individuals to participate in collecting air quality information. One of the developed sensing devices was the Little Environmental Observatory (LEO) developed by Ateknea [53]. It is a prototype with an indicative market price below 500€. The sensor system measures nitrogen monoxide (NO), nitrogen dioxide (NO_2_) and ozone (O_3_) using Alphasense A4 series electrochemical microsensors [54]. The unit contains a built-in ambient temperature (°C) and relative humidity (%) sensors (Sensirion SHT11, Stäfa, Switzerland) in addition to a Pt1000 sensor used for temperature compensation of gas measurements. Table 1 gives the specifications of the Alphasense electrochemical microsensors, while Figure 1 shows the dimensions and the location of the sensors in the device. Depending on the model, the wearer could clip the device on a belt/pocket (weight 164 g) or have it attached to an armband (weight 185 g). The battery lasts up to 20 h depending on Bluetooth usage and sampling frequency. Details regarding testing and performance evaluation of the LEO are given elsewhere [2,55,56]. The testing comprises sensors evaluation in the laboratory against traceable gas standards under controlled ambient conditions, against each other during co-location as well as comparison with stationary air quality monitoring reference data under field conditions.

**Figure 2:**
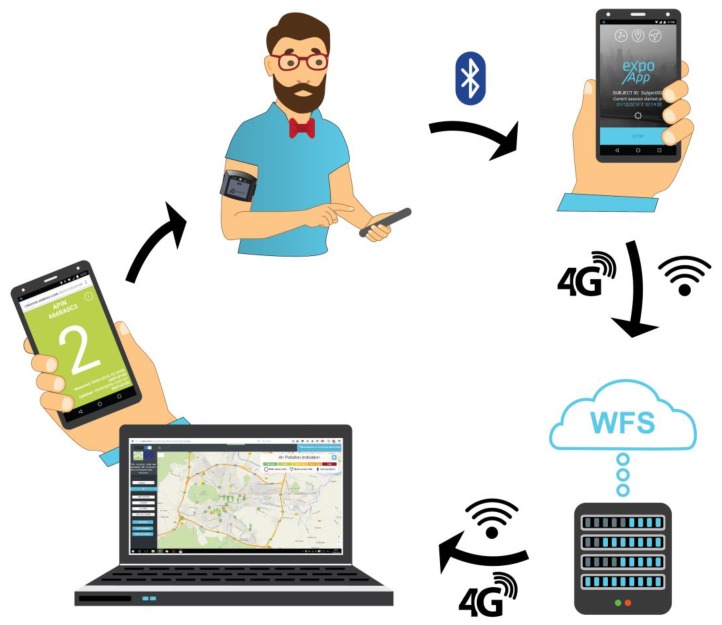
Data flow from the sensor system to the user via a smartphone and the server.

For data collection, the LEO device was paired via a Bluetooth 2.1 connection with an Android smartphone. An app (ExpoApp) allowed the smartphone’s Global Positioning System (GPS) unit to log the user’s geolocation while collecting data. With a 10 s sampling frequency, the smartphone was capable of sending 1121 bytes of data every minute to a Web Feature Service (WFS) server via Wi-Fi or cellular data. A time-stamped signal of the individual sensors, GPS coordinates and accelerometer data were also sent to the server where the data was stored and processed. The data was visualized back to the user in near real time through the ExpoApp app, as a 5 scale Air Pollution INdicator value (APIN). Figure 2 shows the data flow of the system.

The APIN was calculated using all three measured pollutants, using a procedure based on the Common Air Quality Index (CAQI) approach developed as part of the CITEAIRII project [58]. There are important differences between CAQI and APIN. To provide sufficient temporal granularity, APIN uses 1 min averages instead of hourly data, and further, the low-cost sensor data are of unknown- or low-quality [3], while the CAQI is based on air quality monitoring made in compliance with the requirements of EU Directive 2008/50/EC [59].

ExpoApp is also able to display two hours of historical data as time plots of APIN and Activity index. To calculate the Activity index, ExpoApp used the smartphone’s own accelerometer. In addition, the user was able to access an online web visualization tool [60] that shows their own tracks based on GPS data. See also Figure 2. These tracks were only visible to the user who generated them via a unique user ID defined during the pairing phase between the sensor system and ExpoApp. The public was able to see the last location of the units colored according to the last recorded APIN, together with the available data for the city, using project specific web visualization features.

The project team members provided initial feedback about the systems prior to the field campaign, and the necessary changes and updates were made.

### 2.3. Data Analysis

To analyze the UX data, the transcripts from the focus groups and interviews (a 65-paged textual corpus) was coded. This was achieved using mixed coding methods that combined Magnitude coding, Descriptive coding, In Vivo coding, and Recommendation coding in the Evaluation coding method as described in [61]. For traceability, all the entries were numbered both in the textual corpus and in an excel file. The mixed coding process was used as follows; every entry received a main code (Descriptive coding), which emerged during the first round of coding and was accompanied either by a descriptive sentence or a citation (In Vivo coding). Where appropriate, a Magnitude coding indicated whether the feedback was positive (+) or negative (-). Recommendations were tagged with a “REC” (Recommendation coding). A second round of coding followed where similar entries from the first round were further clustered and re-organized.

## 3. Results and Discussion

Twenty-eight categories based on user experience emerged (Table 2). Several entries were also classified under multiple categories, e.g., when volunteers gave feedback with suggestions about ExpoApp, the feedback was categorized both under “app” and “REC”.

The main categories were further modified in the second round (R2) of coding, which meant that several categories from the first round were reclassified, e.g., Behavior, Time, External people, LEO, and Uncategorized. This resulted in 22 categories of UX. We then restructured REC to reflect the target of the recommendation, e.g., hardware and app. In addition, a new category, Data visualization, was created to extend the codes App visualization and Web visualization. Table 2 gives the code frequency for each round. The coded more detailed feedback is available in the Supplementary Materials.

The coding process worked as a tool to form a structured overview for the final evaluation, and many of the main categories complement each other. When writing the manuscript, the final, most aggregated, categorization emerged and comprised the following four themes: (1) User experience, (2) Feedback on the sensor system and its functionalities, and (3) User needs and recommendations and (4) Possibilities.

### 3.1. Structured Feedback

#### 3.1.1. User Experience

The volunteers were positive towards the idea of portable low-cost sensor systems, and the idea that individuals can measure air quality wherever they are. They thought the idea was interesting, nice and beneficial and were interested in having such data. Volunteers thought that crowd-sensing methods are a good way to obtain higher resolution data, and being able to monitor indoor conditions was seen as especially useful compared to static air quality monitoring stations. They also felt positive that such projects exist and hoped that there would be a follow-up study. Some volunteers were surprised to learn that it is possible to measure air quality with such a small device.

Feedback revealed that user-friendliness was one of the main issues. This was not surprising since the device and accompanying software was a prototype designed for researchers rather than for the public, and hence did not meet the needs of the end user (public). In addition, the software was developed by a hobbyist programmer, rather than experienced software developer. Even the more technically oriented volunteers proposed thought ExpoApp needed simplifying. Several volunteers stated that the device in its current form is not ready for citizen science activities. The volunteers felt that the units did not perform well in everyday use, as various technical issues, e.g., the app crashing, having unstable connection and short battery life, kept hindering their use, which reduced the volunteers’ motivation to continue using the device. Many were frustrated that it took a long time to pair the device to the smartphone in the morning when they had the least time (ID1). The volunteers wished for a simpler solution, e.g., a simple start/stop button for continuous data collection. This is in agreement with Thompson [22] who emphasized the need for an effortless interaction in the data collection process.

ID1: “*If it only took few seconds to fix the problem it would be fine. It took a minimum of five minutes, which in itself is already too much. And it usually freezes right when you do not have the time. E.g., when you already have dressed your kids in the winter clothes and are ready to go to the car*”.

UX also highlights issues with ExpoApp, which is a necessary component of the monitoring system. Volunteers were disappointed, frustrated and annoyed with the app, and noted that the phone app did not work properly and did not collect data successfully. Further, they pointed out that, the information provided by the device (categories of APIN) did not match the perceived air quality. Volunteers were also frustrated with the time and effort required to set up the device and to overcome technical issues. Some volunteers were happy to learn how to work with the new technology even if it meant learning to use a force-stop function. Overall, the results reveal that the volunteers expected the system to be user-friendly and older volunteers commented that perhaps, young people might have more time to experiment with a constantly failing technology. Despite this, even after encountering technological issues, several volunteers remained optimistic. Volunteers also sympathized with us about how the sensor system did not work as promised and wished the device could have helped them learn about local air quality.

For many volunteers, their motivation to participate was the idea of experimenting with a new emerging technology and gaining insight into air pollution, especially near their home. They also expected to see spatial differences in air pollution when moving between areas with perceived lower and higher air pollution. They were also interested in monitoring air quality alongside their daily life, while others mentioned that this type of research is important because they want their children to live in a clean environment, and they wanted to check the air quality in their neighborhood. Several volunteers also liked the idea that their participation would contribute to the development of the technology, i.e., towards meeting user needs. These motivations are similar to the findings of Commodore et al. [21], who summarized related studies, and found that the primary motivations behind participation are related to a perceived risk to health, proximity to pollution sources, urban sprawl, living in unmonitored areas, and a desire to know more about air quality. Furthermore, volunteers in our trial emphasized that they were interested in the technology. Hu et al. [12] found that people are more interested in their own personal air pollution exposure than in overall air quality. Our findings were similar, and suggested that people were more interested in personal exposure and air quality in their immediate surroundings, especially near to their home. Hence, it is valuable that mobile devices can collect and receive information about one’s immediate environment.

Once the volunteers began to use the device, technical issues resulted in volunteers becoming demotivated about continuing to use the device, but fortunately this did not affect their desire to fulfil their task, which was to test and evaluate the device. Many of the technical issues encountered were also barriers for further action, for instance, several volunteers stopped using the device after several failed attempts to fix a technical issue, or when fixing the problem took too long (ID12). Five of the volunteers reported using the research prototype only for three to four days instead of the suggested seven days. For many of the volunteers it was not interesting enough to see only one or two APIN values displayed on their smartphones. However, the webpage provided some additional air quality information for their city, based on data obtained from other devices. Accessing an additional webpage to see additional visualization was too much effort for some volunteers.

ID12: “*It crashes already at the settings. When they released a new update after a few days I got excited that perhaps it would work, but it didn’t*”.

Many volunteers pointed out that this device would not be useful or have any benefit in daily life, and that, although the sensors in the device were useful outdoors, people spend a lot of time indoors and so such measurements would not be useful. The device was also considered burdensome, especially since the device needed frequent attention. Several of the volunteers were also unconvinced as to whether or not the device could empower a user to change their behavior, and some could not see the added value in having such a device since “common sense already tells you the time and locations to avoid air pollution” (ID17). The volunteers did believe in the “power of the masses” and suggested that a wide-scale simultaneous deployment was needed to make the data useful for others, rather than just for the individual with the device. There was a belief that substantial actions towards air pollution reductions are needed at the city level, e.g., closing roads, before any changes would be seen on a citywide air pollution map. Nearly all emphasized that such a device must work flawlessly before they are ready for the public. In terms of study length for future studies, one month was considered a suitable time-scale for exposure studies since one month was considered sufficient to capture the diversity of one’s behavior. They believed also that interest in the experiment involving a new gadget would wane within a month and continuing to carry the device would become an obligation. Interestingly, privacy was not a huge issue, albeit volunteers were aware of and agreed to have their movements tracked. They considered it normal in these kinds of projects. Several of the volunteers pointed out that if any data was to be made public, they would not want to be identified or for anyone to be able to know where they live.

#### 3.1.2. Feedback on the Sensor System and Its Functionality

The LEO device received mostly negative feedback, even if some volunteers commented positively about its compactness and how it was a “cool” gadget. They also thought that having an armband as a carrying option was a good solution. Most volunteers were satisfied with the selected air pollutants although several would have liked to have additional parameters and to cover also the indoor spectrum. Most negative feedback was about the device being bulky and clumsy to transport even with the existing attachment options and its tendency to fall to the ground if not attached properly. It was also pointed out that one had to constantly remind oneself to carry it.

When using the device, volunteers had several issues including the time it took for the sensors to stabilize, which resulted in the APIN not changing rapidly enough. Volunteers found this inconvenient, since it meant that they had to remain at one location for a significant length of time to allow the device to adapt to its environment, and that the data was no longer representative when having moved from one location to the next. Also disappointing was that when they expected there to be a difference in air quality, e.g., when moving from a park to a busy street, the device did not register any change in air quality. Moreover, many volunteers were not sure if the device was actually collecting data (ID2), which led to data losses and more disappointment. In addition, due to the app crashing and connection issues between the device and ExpoApp the device had to be frequently reset. This led to data loss—a main source of frustration. The battery life of the hardware lasted longer than the phone battery, yet the volunteers found it ran out of charge quickly, unexpectedly and without warning, hence it was important to remember to charge it regularly. The device also had a blinking LED that indicates data acquisition, Bluetooth connectivity (when established) and the charging state of the battery. Some of the volunteers were bothered by this, especially in the evening in the dark. One volunteer even covered the LED indicators with electric tape.

ID2: “*It would be good if one would know for sure if the device works or not. I know there are those indicating LED lights, but they do not always work the way they should. It leaves the user confused. There should be a clear indication if it works or not*”.

ExpoApp was described as unstable, unreliable, buggy, non-useful, bad, complicated, clunky, non-self-explanatory and slow. Only one volunteer claimed it was easy to use. The volunteers reported that the app constantly kept crashing or froze without any obvious cause, and this often happened when not having attended to the phone for some time, e.g., while sleeping or at work. They also complained that once the app had crashed, it then took several steps and an unreasonable amount of time to get it to work. Sometimes just restarting the app was sufficient, but other times the app had to be force-stopped and the app reinstalled. Data was also lost in various ways, which disappointed the participants. Sometimes data was erased because of having to delete the app, but mainly data was lost because the user thought the device was collecting data, even though it was not. Apparently, it was not obvious when the device was either collecting data or transferring data to the server. The data connection was also said to be poor. Curto et al. [62] also reported various data gaps, e.g., missing values or repeated zeros with various other low-cost sensor systems which are already on the market, which opens a discussion on how to reduce the failure or malfunction rate of such devices. Data connection issues also resulted in ExpoApp crashing multiple times a day, which meant that the volunteers had to create multiple usernames. This became an issue when a volunteer wanted to access the data portal retrospectively, who then had to remember all of their user names and associated dates. This led to them not being able to access all of their data.

Volunteers also experienced various Bluetooth connection issues. The connection between the sensor system and the phone was difficult to establish and maintain. For this reason, the phone and the hardware needed to be kept in close proximity, but unfortunately not everyone remembered this all the time. The connection was supposed to be automatic once the phone and the sensor system unit were in close proximity, but this was not always the case, which caused ExpoApp to freeze. In addition, some of the volunteers could not participate because the app was only available on the Android operating system, and in some cases even with an Android phone, the app would not work. This left volunteers frustrated because they were promised that it would work on a certain version of Android regardless of the phone’s make. Volunteers also commented that overcoming these constant technical issues with ExpoApp would be even more challenging for a less technically minded person. Battery life was also an issue. Not only did the GPS and Bluetooth connection rapidly drain the phone’s battery, but the device kept shutting-off unexpectedly because there was no clear indication of battery life.

The data itself was considered interesting and many volunteers would have liked to discover more about their immediate surroundings and the places they frequently visit. For some volunteers, the five scale APIN color codes were sufficient, while others would have preferred a much finer-scale and would have like to see the values actually changing. This finding agrees with that of Zappi et al. [36], where the EPA color guideline and number also did not provide a sufficient level of detail for the participants. Many volunteers in our study would have liked to see numeric data and graphs of individual pollutant concentrations rather than an index. In a similar initiative performed by Bales et al. [37], air quality was visualized as an air quality index while a more detailed analysis was provided for more involved users.

Calibration issues means that data from low-cost sensor systems show mostly either relative values (higher and lower pollution levels) or an aggregated level of air quality [3], which raises questions whether showing numeric data to the end users will be done in the near future (also see reference [1]). Data quality on its own was rarely a topic of discussion, but a lengthy sensor stabilization time significantly lowered their trust in the measurements making the device ineffective as a mobile unit. Questions also arose concerning the quality of the data, noting that it differs from official data, and whether it makes sense to calculate the APIN using data obtained from low-cost sensor systems. There were also opposing views about whether increasing the number of low-cost air quality sensor systems in a city for modelling purposes would result in better data.

The web visualization portal was mainly perceived positively and described as being OK, nice, interesting, and that it was easy to use and navigate. Some volunteers did not see any need for improvements, yet others would have liked the ability to examine individual pollutants and their concentrations instead of having just an index value. Unfortunately, some volunteers were using the portal when updates took place and reported how the portal was unreliable, while others did not know that they can display multiple user tracks. It was also not obvious that they needed to enter the user ID from the app, and not the hardware ID.

#### 3.1.3. User Needs and Recommendations

The volunteers’ recommendations reflect the user needs of low-cost portable air quality sensor systems. All volunteers made recommendations concerning the used sensor system as well as general recommendations for future low-cost portable sensor systems throughout the focus group discussions. These included the fact that volunteers would prefer a self-explanatory device, but if it did require a user manual, they would like it to be clear, simple, concise and accurate: on demand technical support was also deemed for future studies. The volunteers also thought the hardware should have multiple transport options and that the device should be much smaller than the one used in the study. Many volunteers suggested integrating the sensors in a device that a user would be already carrying with them, such as a phone or a smart watch. Other suggestions included having additional sensors, like particulate matter (PM), carbon dioxide (CO_2_), and that the device should be able to detect and display differences in air pollution concentrations. Temperature and humidity are also parameters of interest. However, they were not visualized in the used sensor system despite them being part of the sensor unit, as they were only used for temperature compensation of gas measurements. Bluetooth and GPS issues should be solved and battery life maximized, e.g., by using low energy Bluetooth. Other suggestions included having the data frequency set automatically to a lower sampling rate and the GPS and Bluetooth in stand-by mode when the person is static. Another suggestion was to send data to the server at least twice per day to avoid having to be constantly connected to the 3G network with an option to require real time data on demand. In addition, there should be a battery level indicator either on the device or in the app. Some volunteers expressed a wish to control the hardware.

Volunteers also expressed a desire for information on exposure and on the spatial distribution of air pollution. They suggested developing an app that is more intuitive, self-explanatory, simple and user-friendly than the use one, meaning a device that is “good to go” and needs as little button pressing as possible. Having only an on/off button was suggested. In addition, Bluetooth, GPS and 3G could be turned on automatically rather than manually, and for it to be obvious when the device is collecting or sending data to avoid any misunderstandings and to prevent data loss (ID17). A smartphone app should also be made for iOS to include more volunteers. It was also suggested that the app could have other functions, such as, a function which would alarm the user when air pollution is too high. In order to have context for the data, the volunteers suggested a function that would tag and add frequently visited locations and activities, e.g., home, work, exercise, etc. They also wanted to be able to use the same user ID upon each restart of the device, with the option to change it if they wish. The option to have a phone app that combines subjective air quality and measured objective values was raised multiple times. For example, the user would indicate their subjective estimation of air quality at their location followed by a verification of the current measured air quality.

ID17: “*I would like that there were as few steps as possible. That it would be automatically connected and sending the data. There needs to be as little such extra pressings of buttons like “OK”, “save”, “confirm” etc. It would be good that once you press the stop button, you would get a notification that you had been measuring successfully. That you get a feeling everything went well*”.

To improve the data visualization experience, volunteers suggested having mobile friendly data visualization similar to the web visualization. More granularity was also desirable, since with the used system (5 scale APIN) the values did not vary sufficiently to be of interest e.g., (ID3). Many said they would prefer to see individual pollutant concentrations with indicative limit values. Also, access to historic data of the user’s tracks is considered important, but the visualized data should be anonymized.

ID3: “*I was disturbed by the fact that it only showed number 3 all the time, regardless where I went*”.

Considering that the used device was not collecting data autonomously or being used constantly, the volunteers suggested it would benefit from having a reminder function. Kefalidou and Sharples [63] recommends that users are reminded to use a device with a pop-up function, but such reminders should be kept to a minimum. The volunteers agreed that the appropriate length to carry such device is one month, and also implied that they were not ready to integrate it permanently into their daily lives. However, if the device was much smaller, like the size of a wristwatch, they could see themselves using it more frequently. According to a recent review on wrist wearable devices, current air quality sensor technology is not sufficiently mature and hence, is not yet part of mainstream wrist wearable devices [64].

Finally, the volunteers suggested the price for a similar commercial device should be under 100€, although some would pay 150€, but 200€ was considered too expensive.

#### 3.1.4. Possibilities

The volunteers brought up various options and suggestions regarding what could be done in the future with low-cost portable air quality sensor systems. Similarly, Barzyk et al. [65] collected Newark’s Ironbound community’s suggestions on what would be the next steps with regards to sensor technologies. The volunteers in our study suggested that such devices have the potential for raising awareness about the spatial distribution of air pollution, which could affect preferred routes and places to spend time and live in, and hence, improve one’s quality of life. Such devices could be used also for monitoring indoor air quality, e.g., in the home and in the car. Crowd sourcing projects, industrial air pollution monitoring and estimating property values were some of the applications suggested. Vulnerable groups, such as asthmatics, were seen as possible target groups who could receive information about their exposure from such devices. In addition, city authorities, nongovernmental organizations and kindergartens were listed along with schools that could use them to perform various experiments. Ideas about attaching the devices to city bikes or busses, or google street-view cars were raised. Having such data publicly available was seen as self-evident, and the city authorities were seen as the most obvious contact point to make data available. The volunteers also mentioned how such devices could open up discussions with polluters and decision makers. In order to reduce costs for the end users of low-cost portable air quality sensing systems, a rental service operating through either a company or a research institute for individuals and schools was suggested. A sensor loan program for public use has already been suggested by Barzyk et al. [65]. Moreover, volunteers expressed their interest in wanting to know about the air quality in their own neighborhood, and wished for such devices to be used to determine where the air quality is poor and how to improve it.

### 3.2. Redesign of the Low-Cost Sensor System for Air Quality

During the field campaign, running feedback was provided to the hardware and web developers, pointing out the issues that the volunteers were having. At the end of the campaign, the feedback from the interviews and the focus groups was aggregated, and sent to the development team. The volunteers’ feedback contributed to a new version of the hardware (see reference [66]). The resulting unit, called the Ateknea Air City monitor (AACM), is half the size of the original unit used in this study, has a lower energy draw Bluetooth connectivity, and improved battery life, and is one-step closer towards meeting the end-user needs. The field evaluation of the AACM occurred at the end of the CITI-SENSE project and neither new software nor visualization tools were implemented during the project, nor was the new sensor system tested afterwards.

### 3.3. Limitations

This study had several limitations. For example, by gathering volunteers’ feedback retrospectively and relying solely on what they reported, might have led to details getting lost on how the volunteers operated the sensor system. During the focus group sessions, certain volunteers dominated the discussions and this may have prevented other volunteers from voicing their opinions. The profile of the volunteers group was not reflective of the general population, and the volunteers were clearly more research minded and technically capable than a cross sample of the public. In contrast, the profile of the volunteers in this study might well represent the reality of a possible citizen scientist profile. Our recruitment goal was to obtain feedback from people outside of the project and our institute. Moreover, any generalizations are based on volunteers’ evaluation of a single prototype sensor device with an associated phone application. Hence, many more portable low-cost air quality sensor systems should be studied in the future to evaluate them from the point of view of the public. Moreover, the end users should be included in the earlier design of the sensor system [46]. The lack of earlier end user involvement in the design process in our case study highlights the importance of doing so. Malfunctioning of the sensor system was an issue, and in some cases prevented the seamless collection of data and meant that several volunteers could not use the device to its fullest potential. Questionable data quality from low-cost sensors [67] can also be seen as a limitation. Finally, since the translation and coding of the interview data was done only by one person, which can have led to subjective bias. Despite these limitations, based on personal communication with other project investigators the authors believe that the results present an accurate representation of UX.

## 4. Conclusions

This paper evaluates UX with respect to a prototype of a low-cost portable air quality sensor system from a citizen scientists’ perspective and provides user experience feedback for future developers. The sensor system comprises a sensor device, accompanying phone app and web visualization. Due to the issues related to the app design, e.g., freezing, crashing and having connection issues, the app received more comments than any other components of the system. Our paper brings insights into user experience with a sensor system which was considered ready for public, yet, as it lacked user involvement in an earlier state, the collected feedback had a negative tone and various necessary improvements were suggested. Consequently, we were able to map the user needs of future low cost-portable sensor systems. Personalized air quality information based on data from low-cost portable sensor systems can potentially help people to make informed decisions on their exposure. For this reason, this technology should be designed with and for the public.

By including volunteer feedback, this study uncovered issues relating to usability that commonly used methods of self-evaluation by project members could not provide. Our results showed that the tested low-cost portable sensor system was perceived as non-user friendly and not ready to be widely deployed by end users, even though comments provided by the project team members were taken into account by the device developers prior to the field campaign. The feedback from volunteers highlighted meaningful changes and features, which can be used to improve the next generation of such devices and contribute to understanding the limitations and opportunities of technology that is fit for purpose.

The root cause of the negative feedback can be associated with the poor operational performance of the phone app, while most of the positive feedback of the sensor system related to its potential. The prototype sensor system used in this study frequently complicated the volunteers’ lives: they desired a simpler, less visibly prominent, and, not least, a more reliable device with additional visualizations. They especially did not like the results to be in conflict with their perceived air quality. Key recommendations are as follows:

- Involve potential end-users early in the development phase in iterative cycles;
- The device should be “good to go” providing results immediately when activated;
- Minimal effort to operate with a simple start-stop button;
- Using the device should be self-explanatory and intuitive;
- It should come with a functional and user-friendly smart phone app capable of running on different mobile operating systems (iOS, Android, Windows, etc.);
- The app should display near real-time data with reliable and stable data transmission;
- The device needs to indicate successful data collection and battery levels;
- The device should detect and visualize differences in air quality at a higher resolution than the 5-level scale APIN which is too coarse to be interesting;
- The sensor system should provide a visualization which is in agreement with users perceived air quality i.e., indices might mask out individual high air pollutant concentrations which the user detects;
- The visualization should be tailored and adapted for different user groups, e.g., to include different levels of visualization for basic and advanced users;
- Users would prefer to see individual pollutant concentrations with indicative limit values;
- Default visualization should include present and past data linked to the user’s location.
- Smartphone apps should be developed by professional mobile app developers.

Despite the negative feedback, it was evident that the volunteers liked the idea of a portable low-cost air quality sensor device and could see the potential of such a device, albeit there remains significant room for improvement. The volunteers in this study were optimistic about the future of the technology, even though the prototype device did not work as they hoped. In short, the users remain interested as long as the technology works. To keep costs down, a rental option should also be made available for individuals and interested groups such as schools. This would satisfy the need to explore and use the device only as long as the user remains interested.

This study illustrates that initiatives, such as citizen science projects, aimed at improving data availability (coverage, temporal resolution, value to users) should not only focus on improving data quality, but also UX. This case study demonstrates that it is not enough simply to test a prototype amongst project team members, since end users will highlight the most pressing design faults which need further development prior to the device being ready for wider deployment. Therefore, the authors recommend testing prototypes of low-cost portable air quality sensor devices with a small number of volunteers prior to data sampling campaigns, if their purpose is to go beyond proof of concept.

## Figures and Tables

**Table 1:** Specifications of the Alphasense electrochemical microsensors [57].

Parameter	Sensor Name	Operational (Measurement) Range	Response Time	Diameter	Weight
NO	NO–A4	0–20 ppm	<25 s	20.2 mm	<6 g
NO_2_	NO2–A42F	0–20 ppm	<60 s	20.2 mm	<6 g
O_3_	OX–A421	0–20 ppm	<45 s	20.2 mm	<6 g

**Table 2:** Number and frequency of codes in each coding iteration (coding rounds R1 and R2).

	Main Codes	R1/R1 f Total	*R1: REC*	R2	*R2: REC*	R2 f Total
	Recommendation (REC)	84	*100*	45	*56*	103
	App	50	*35*	27	*20*	53
	Barriers for further action	43	*3*	15	*3*	45
	Possibilities (if improved)	37		21		45
	Hardware	24	*17*	16	*9*	27
	Motivation	22		14		25
	Positivity about the general idea	18		8		21
	Data loss	14	*3*	5	*1*	18
	Web visualization	13	*5*	9	*2*	16
	General user experience	13		9		13
	Expectations	7		4		8
	Privacy	7	*2*	2	*1*	7
	Quitting (reasons)	6		4		7
	Battery	7	*5*	2	*4*	7
	Length of use	4		4		6
	Data connection	6	*5*	2	*3*	6
	App visualization	3	*2*	4	*2*	4
	Data quality	4	*1*	4	*1*	4
	GPS	4	*6*	3	*4*	4
	Optimal participation time	4	*3*	1	*1*	3
	Product price	2	*2*	3	*1*	3
	Behaviour	3		^1^		
	Time	10		^2^		
	Data visualization		*8*		*4*	
	External people	2		^3^		
	LEO	26	*3*	^4^		
	CityAir	25		^5^		
	Uncategorized	5		^6^		
Total	28	443	*100*	202	*56*	425

^1^ Under “Possibilities” in R2; ^2^ Mostly covered by “Barriers for further action” in R2; ^3^ under “Possibilities” in R2; ^4^ covered by other categories in R2; ^5^ Excluded as it is outside the scope of this paper, pertaining to another data collection tool used in CITI-SENSE; ^6^ categorized in R2.

## Supplementary Materials

The following are available online at http://www.mdpi.com/1424-8220/18/11/3768/s1, Table S1: Coding round 1; Table S2: Coding round 2; Table S3: Additional coding iterations for “REC” in R1; Table S4: Additional coding iterations for “REC” in R2; Table S5: Statistics of “REC”; Table S6: Code frequency statistics; Table S7: Volunteers statistics.
